# Can a Teaching Programme Help Reduce Radiation Exposure During Extra-Capsular Proximal Femur Fracture Fixation?

**DOI:** 10.7759/cureus.96585

**Published:** 2025-11-11

**Authors:** Owen Mitchell, Joel Xavier, Qamar Mustafa, Mohamed Mahmoud, Rebecca Mills

**Affiliations:** 1 Trauma and Orthopaedics, Dorset County Hospital NHS Foundation Trust, Dorchester, GBR; 2 Trauma and Orthopaedics, Dorset County Hospital NHS Foundation Trust, Dorchester , GBR

**Keywords:** dynamic hip screw fixation, hip and proximal femur trauma, orthopaedics & traumatology, proximal femoral nail, radiation dose reduction

## Abstract

Background

Proximal femur fractures (PFFs) represent a major healthcare burden in the United Kingdom, with approximately 76,000 cases annually. Extra-capsular fractures are commonly treated with Dynamic Hip Screws (DHS), short or long intramedullary nails (IMNs), all of which require intraoperative fluoroscopy. Consequently, orthopaedic surgeons and patients are exposed to ionising radiation and the associated stochastic and deterministic risks. Despite regulations under the Ionising Radiation (Medical Exposure) Regulations 2017 (IR(ME)R), a wide variation in radiation safety knowledge persists, and there are no established benchmarks for acceptable exposure. This audit aims to assess whether a departmental teaching programme could reduce radiation exposure during extra-capsular PFF fixation and to explore differences between consultant and trainee-led cases.

Methodology

This audit was conducted at Dorset County Hospital, United Kingdom, audit number 6323. Patients with closed extra-capsular PFFs managed using DHS, short or long IMN fixations were included. Radiation exposure, expressed as dose-area product (DAP), was recorded from the Picture Archiving and Communication System (PACS) across two audit cycles: before and after delivery of an educational programme, emphasising radiological stewardship and safety. Statistical analysis was performed using independent t-tests, with significance set at *P* < 0.05 and effect sizes reported using Cohen’s *d*.

Results

A total of 39 operations were analysed pre-intervention and 31 post-intervention. Overall mean radiation exposure decreased marginally from 347.4 to 344.6 cGycm² (*P* = 0.97, *d* = 0.01). Reductions were observed for DHS, from 245.6 to 210.8 cGycm² (*P* = 0.40, *d* = 0.27) and short IMN fixation, from 605.9 to 375.9 cGycm² (*P* = 0.18, *d* = 0.67). Long IMN fixation demonstrated a statistically significant increase in mean dose, from 246.8 to 764.5 cGy·cm² (*P* = 0.01, *d* = -2.56). Consultant-led cases showed a non-significant increase in mean radiation dose from 404.0 to 462.6 cGycm² (*P* = 0.75, *d* = -0.16). Cases led by other grades demonstrated a small, non-significant reduction from 332.8 to 303.6 cGycm² (*P* = 0.67, *d* = 0.12).

Discussion

The teaching programme was associated with modest improvements in radiation exposure for DHS and short IMN fixation, suggesting that structured education may enhance radiological stewardship. However, the increase observed in long IMN fixation likely reflects case complexity and limited sample size rather than a failure of the educational intervention. These results reinforce that educational measures alone are insufficient; lasting improvements require integration with simulation training, feedback on fluoroscopy use, and adherence to optimised imaging protocols.

Conclusions

A single departmental teaching session produced non-significant reductions in radiation exposure for DHS and short IMN procedures, supporting the role of targeted education in improving radiological practice. The increase in exposure for long IMN fixation likely reflects procedural complexity rather than educational failure. This highlights the need for continuous training, technical optimisation and regular audit to ensure adherence to IR(ME)R principles. Larger multicentre studies with case-mix adjustment are warranted to establish national reference levels and to evaluate the sustained impact of educational interventions on radiation safety.

## Introduction

Proximal femur fractures (PFFs), often termed hip fractures, represent a major healthcare burden in the United Kingdom, with around 76,000 hip fractures occurring annually across the United Kingdom [1]. A recent population-based study from England spanning 2014-2024 reported 704,762 hip fracture hospital presentations among adults aged ≥50 years, with incidence rates declining slightly pre-COVID but rising above expected levels in the post-pandemic period [1]. These fractures predominantly affect older, frail patients and are associated with high morbidity, mortality, prolonged hospitalisation, and substantial economic cost [2].

PFFs are usually classified into intra-capsular and extra-capsular fractures. Surgical intervention is the standard treatment for extra-capsular PFFs, with intramedullary femoral nails (IMNs) and dynamic hip screws (DHS) being the most commonly employed fixation methods in the United Kingdom. IMNs are favoured for delivering on-axis fixation, contributing to biomechanical stability, and are particularly beneficial in highly comminuted, reverse oblique and subtrochanteric fractures. The DHS is commonly used to treat stable intertrochanteric fractures because it allows controlled collapse and compression at the fracture site as the patient begins weight-bearing [1,3].

In the United Kingdom, DHS fixation and IMN fixation are often among the first major orthopaedic procedures that junior surgical trainees are expected to learn and assist with [4]. These operations, performed for extracapsular proximal femoral fractures, introduce trainees to fundamental surgical principles such as fracture reduction, guidewire insertion, and implant positioning.

Intraoperative fluoroscopy is essential during these procedures to ensure accurate fracture reduction, guidewire placement, reaming depth, screw insertion and final implant positioning. However, this imaging exposes both patients and surgical staff to ionising radiation. Overexposure can lead to *deterministic* effects, such as skin injuries (erythema, epilation and tissue necrosis) and *stochastic* effects, leading to a cumulative increased lifetime risk of cancers. Operating room staff, who may be exposed to radiation over extended periods, are at particular risk [3,5].

Recent studies have highlighted factors contributing to increased radiation exposure during PFF fixation. A study by Elbahi et al. identified that complex fracture patterns, prolonged surgical times, and the use of multiple fluoroscopic views significantly increase radiation dose-area product (DAP) and screening time [3]. Additionally, a survey of UK orthopaedic surgeons revealed a lack of knowledge regarding radiation safety practices, with many reporting inadequate training and inconsistent use of personal protective equipment (PPE) [5]. Junior surgical trainees may initially rely heavily on intraoperative fluoroscopy, resulting in frequent imaging and prolonged exposure to ionising radiation [6].

The Ionising Radiation (Medical Exposure) Regulations (IR(ME)R) 2017, as amended in 2024, mandate that all medical exposures involving ionising radiation must be justified and optimised [7]. The ALARP (As Low As Reasonably Practicable) principle underpins these regulations, requiring that radiation doses be kept to the minimum necessary to achieve the desired clinical outcome [7]. Compliance with IR(ME)R is essential to safeguard patients and healthcare professionals from the potential harms of ionising radiation [5,7]. However, despite adherence to the ALARP principle, there are currently no proposed guidelines or targets for maximum radiation dosage during these procedures. Additionally, it has been shown that more experienced surgeons tend to use less radiation during these procedures than their junior colleagues [8].

The primary aim of this audit was to try and reduce radiation exposure during DHS and IMN operations for extra-capsular PFFs through an educational programme. The secondary aim was to compare the radiation exposure during cases led by consultant surgeons with cases led by more junior surgeons to investigate if less experienced surgeons used more radiation.

## Materials and methods

This study was conducted at the Department of Trauma and Orthopaedic Surgery at Dorset County Hospital, located in Dorchester, United Kingdom. The approval for the audit was granted by the Clinical Audit Department of Dorset County Hospital in 2025 (audit number 6323).

An initial audit was performed to establish current practices within the Department of Trauma and Orthopaedic Surgery. The inclusion criteria comprised patients of any age who presented with closed extra-capsular proximal femur fractures, including basicervical, intertrochanteric, and subtrochanteric fractures (defined as fractures of the proximal femur up to 2 cm distal to the lesser trochanter), managed with either a DHS or anterograde IMN (long or short) [9]. The exclusion criteria omitted any patients presenting with femoral shaft fractures (defined as fractures of the diaphysis more than 2 cm distal to the lesser trochanter), any revision procedures, and any retrograde IMN procedures [10].

Data were obtained from locally collected National Hip Fracture Database (NHFD) records, which included the patient’s age, the surgical intervention performed (DHS, long IMN, or short IMN), and the grade of the primary operating surgeon. The radiation exposure was then obtained from the DAP report for each patient, which had been published on the local Picture Archiving and Communication System (PACS). The DAP or mean radiation dose describes the amount of energy delivered by the fluoroscopy machine.

Data were collected retrospectively from the Orthopaedic Department’s NHFD for the period of 01 April 2025 to 31 May 2025.

A teaching programme was then delivered to the orthopaedic Consultants, Registrars and Resident Doctors at a local education afternoon. This included audit data collected from the first round of data collection and aimed to emphasise the importance of radiological stewardship, minimising the amount of radiation exposure where possible and the ALARP principle.

The hospital’s data was then re-audited with the same inclusion and exclusion criteria for the time period of 01 July 2025, 31 August 2025. Comparison of the data was then completed to see if there was any statistical difference in the radiation exposure during these procedures before and after the teaching programme. The means were calculated, along with the standard deviation, to calculate the *P*-value and Cohen’s *d* effect.

## Results

Following the application of the inclusion and exclusion criteria, 39 eligible patients were identified. They had a mean age of 82.8 years, and 64.1% of the patients were female. Twenty-one (53.9%) patients were treated with a DHS, 11 (28.1%) with short IMN and 7 (18.0%) with long IMN. The overall mean radiation dose was 347.4 cGycm² per operation. The largest radiation dose was found to be during short IMN procedures, with a mean of 605.9 cGycm², the second largest was long IMN with 246.8 cGycm², and finally, DHS averaged 245.6 cGycm². This audit also found that consultant-led operations had a higher average radiation dose per operation when compared with operations led by more junior surgeons, 404.0 cGycm² compared to 332.8 cGycm² (Table 1).

**Table 1:** Pre-intervention retrospective data gathered for mean radiation dose for dynamic hip screw, short intramedullary nail and long intramedullary nail procedures.

Operation type	Number of operations	Mean radiation dose (cGycm²)	Consultant-led operations, *n* (%)	Mean radiation dose for consultant-led operations (cGycm²)	Other grade-led operations, *n* (%)	Mean radiation dose for other grade-led operations (cGycm²)
Dynamic hip screw	21 (53.9%)	245.6	1 (12.5%)	198.6	20 (64.5%)	248.0
Short intra-medullary nail	11 (28.1%)	605.9	3 (37.5%)	661.4	8 (25.9%)	585.1
Long intra-medullary nail	7 (18.0%)	246.8	4 (50%)	262.4	3 (9.6%)	226.1
Total	39	347.4	8	404.0	31	332.8

After the departmental teaching programme was delivered, the same inclusion and exclusion criteria were applied, which yielded 31 patients. There was a slightly younger age, with the mean being 79.8 years, and 71.0% of the patients were female. Twenty (64.5%) patients were managed with a DHS, 5 (16.1%) with a short IMN and 6 (19.4%) with a long IMN (Table 2). Following the departmental teaching programme, the overall mean radiation exposure decreased slightly from 347.4 to 344.6 cGycm² (*P* = 0.97, *d* = 0.01), indicating no statistically significant or clinically meaningful overall difference.

**Table 2:** Post-intervention retrospective data gathered for mean radiation dose for dynamic hip screw, short intramedullary nail and long intramedullary nail procedures.

Operation type	Number of operations	Mean radiation dose (cGycm²)	Consultant-led operations, *n* (%)	Mean radiation dose for consultant-led operations (cGycm²)	Other grade-led operations, *n* (%)	Mean radiation dose for other grade-led operations (cGycm²)
Dynamic hip screw	20 (64.5%)	210.8	4 (50.0%)	187.3	16 (69.6%)	216.7
Short intra-medullary nail	5 (16.1%)	375.9	1 (12.5%)	215.0	4 (17.4%)	416.2
Long intra-medullary nail	6 (19.4%)	764.5	3 (37.5%)	912.3	3 (13.0%)	616.7
Total	31	344.6	8	462.6	23	303.6

**Figure 1:**
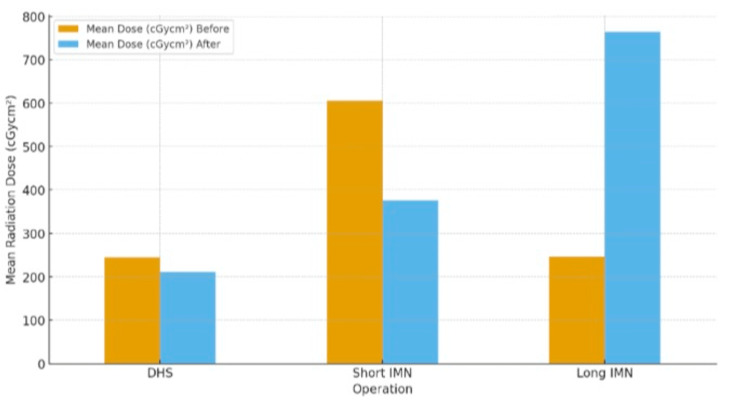
Mean radiation dose (cGycm²) by operation type (dynamic hip screw (DHS), short intramedullary nail, long intramedullary nail). Following the teaching intervention, mean exposure decreased for DHS and short IMN procedures but increased for long IMN fixation. IMN, intramedullary nailing

When analysed by procedure type, reductions were observed for DHS and short IMN fixations. Mean exposure for DHS fell from 245.6 ± 158.8 to 210.8 ± 95.1 cGycm² (*P* = 0.40, *d* = 0.27), while short IMN fixation decreased from 605.9 ± 440.2 to 375.9 ± 201.4 cGycm² (*P* = 0.18, *d* = 0.67) (Figure 1). Although neither change reached statistical significance, the small-to-moderate effect sizes suggest a potential clinically relevant improvement following the teaching intervention (Table 3).

**Table 3:** Mean, standard deviation, P-values and Cohen's d effect size.

Operation	Number of procedures - pre-intervention, *n* (%)	Mean Radiation exposure (cGycm^2^) - pre-intervention	Standard Deviation - pre-intervention	Number of procedures - post-intervention, *n* (%)	Mean radiation exposure (cGycm^2^) - post intervention	Standard deviation - post intervention	*P*-values	Cohen's *d* effect size
DHS	21 (53.9%)	245.6	158.8	20 (64.5%)	210.8	95.1	0.40	0.266
Short nail	11 (28.1%)	605.9	440.2	5 (16.1%)	375.9	201.4	0.18	0.672
Long nail	7 (18.0%)	246.8	168.0	6 (19.4%)	764.5	231.1	0.01	-2.563
Overall	39	347.4	309.3	31	344.6	259.6	0.97	0.01

In contrast, long IMN fixation demonstrated a marked increase in radiation exposure, increasing from 246.8 ± 168.0 cGycm² pre-intervention to 764.5 ± 231.1 cGycm² post-intervention (*P* = 0.01, *d* = -2.56), representing a statistically significant and large negative effect. This likely reflects procedural complexity and limited sample size rather than a true deterioration in practice.

**Figure 2:**
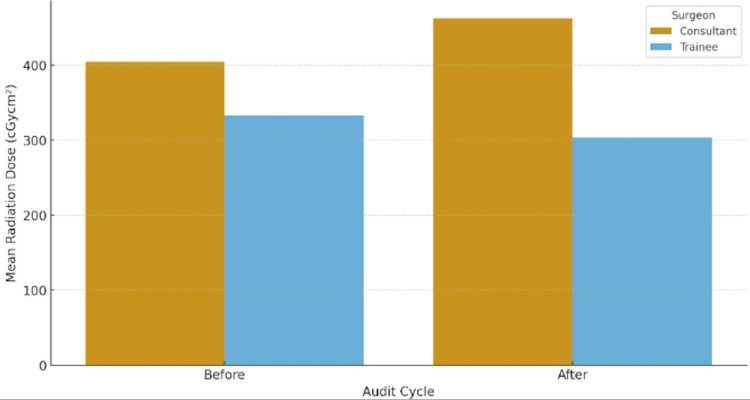
Comparison of the mean radiation dose by surgeon grade (consultant vs. trainee) before and after the teaching programme. Consultant-led cases consistently demonstrated higher mean exposure, likely reflecting greater involvement in technically complex operations.

Comparing surgeon grades, consultant-led cases showed a non-significant increase in mean radiation dose from 404.0 ± 356.7 to 462.6 ± 386.3 cGycm² (*P* = 0.75, *d* = -0.16) (Figure 2). Conversely, cases led by other grades demonstrated a small, non-significant reduction from 332.8 ± 300.6 to 303.6 ± 193.9 cGycm² (*P* = 0.67, *d* = 0.12) (Table 4).

**Table 4:** Statistical analysis comparing consultant-led and other grade-led operations.

Operation	Number of procedures - pre-intervention, *n* (%)	Mean radiation exposure (cGycm^2^) - pre-intervention	Standard deviation - pre-intervention	Number of procedures - post-intervention, *n* (%)	Mean radiation exposure (cGycm^2^) - post-intervention	Standard deviation - post-intervention	P-values	Cohen's *d* effect size
Consultant	8 (20.5%)	404.0	356.66	8 (25.8%)	462.6	386.34	0.75	-0.16
Other grades	31 (79.5%)	332.8	300.64	23 (74.2%)	303.6	193.87	0.67	0.12

Overall, these findings indicate that while the teaching programme produced modest improvements in radiation exposure for simpler fixation techniques, significant reductions were not consistently achieved, and long IMN cases remained associated with higher doses.

## Discussion

This audit investigated whether a departmental teaching programme could reduce radiation exposure during extra-capsular proximal femur fracture fixation. The overall radiation dose per operation fell only slightly after the intervention, from 347.4 to 344.6 cGycm². More notable reductions were seen in DHS and short IMN procedures, whereas long IMN operations demonstrated an unexpected increase.

The reductions observed in DHS (245.6-210.8 cGycm²) and short IMN fixations (605.9-375.9 cGycm²), although not statistically significant, do support the hypothesis that simple educational measures can improve intraoperative radiological stewardship. The structured teaching sessions likely encouraged surgeons to be more deliberate with C-arm positioning, limit unnecessary images, and plan reductions before screening. This is in line with prior studies, which have shown that relatively modest interventions, such as awareness training or simulation-based practice, can alter operative behaviour and reduce radiation exposure [3,8,10]. Such improvements are aligned with IR(ME)R requirements, which emphasise justification and optimisation of all medical exposures in line with the ALARP principle [7].

By contrast, long IMN fixation showed a marked, statistically significant increase in mean radiation dose (246.8 to 764.5 cGycm²). This finding reflects the inherent technical challenges of long nail placement, where accurate positioning of distal locking screws often demands repeated fluoroscopic checks [8]. This finding is reinforced by the scientific literature, which has confirmed that IMN procedures, particularly long nails, are associated with higher fluoroscopy times and radiation doses compared with DHS [9,11]. It is possible that increased case complexity, a small number of long IMN operations, or allocation of technically demanding cases to senior staff amplified this trend. This highlights that while education is important, technical factors and implant choice remain dominant determinants of radiation exposure.

The observation that consultant-led operations were initially associated with higher doses (404.0 vs 332.8 cGycm² for junior-led cases) is intriguing. Previous literature generally suggests that experience correlates with reduced fluoroscopy time and dose [8,11]. In this audit, consultants may have been disproportionately involved in complex fractures, which inherently required greater imaging. Alternatively, consultants may adopt a lower threshold for confirming implant placement radiologically, leading to increased screening despite shorter operative times. This underlines the importance of accounting for case-mix and complexity when comparing radiation metrics between surgeon grades.

When the mean radiation dose is divided by the irradiated area, the entrance skin dose (ESD) is calculated [12]. While the ESD values observed in our cases are significantly below the established thresholds for deterministic radiation effects, adherence to the principles of radiological stewardship remains imperative to ensure radiation exposure is maintained in accordance with established safety standards and regulatory guidance.

Recent national surveys provide important context for this audit. The Radiation in Orthopaedics (RIO) study found wide variation in radiation protection knowledge and practice across the United Kingdom, with poor awareness of ALARP and inconsistent PPE use, particularly among junior surgeons [11]. A 2025 follow-up survey confirmed that these gaps persist despite regulatory frameworks [13]. The BOA has since emphasised the need for structured education and monitoring to mitigate occupational risks [14]. Together with our findings, these publications reinforce the importance of ongoing education rather than one-off interventions.

Cumulative occupational exposure remains a significant concern. Hurley et al. demonstrated that cumulative whole-body doses for orthopaedic teams may approach recommended limits in high-volume trauma settings [15]. Even small reductions achieved through targeted teaching, as seen in this audit, could provide meaningful long-term benefits for staff safety. For patients, minimising exposure is equally important, as stochastic risks such as carcinogenesis increase with cumulative dose [2].

Overall, this audit supports the use of educational programmes as a feasible, low-cost intervention to improve radiation safety during PFF surgery. However, variability in results across implant types and operator grade indicates that education should be embedded within a broader strategy. This may include simulation training, regular feedback of surgeon-specific exposure data, optimised fluoroscopy settings (e.g., pulsed or low-dose modes), and consideration of national diagnostic reference levels to standardise practice.

Limitations

This audit has several limitations. The sample size was small, particularly for long IMN cases, limiting statistical power and increasing the influence of outliers. Patient and case factors known to influence radiation use, such as body mass index (BMI), fracture complexity and intraoperative technical variations, were not controlled for [3,13]. The short follow-up period after the teaching intervention limits the assessment of the sustainability of improvements. In addition, the observational design without randomisation or a control group restricts causal inference.

## Conclusions

This audit demonstrates that a targeted departmental teaching programme can promote greater awareness of radiation safety and modestly reduce radiation exposure during extra-capsular proximal femur fracture fixation, particularly for DHS and short IMN procedures. Although these reductions did not reach statistical significance, the observed small-to-moderate effect sizes suggest a potentially meaningful clinical benefit, especially if reinforced through sustained education and feedback.

Conversely, radiation exposure increased significantly during long IMN fixation, likely reflecting the greater technical demands and case complexity associated with this procedure rather than a failure of the educational intervention. These findings highlight that while education improves awareness and can influence behaviour, it should be complemented by practical measures such as simulation-based training, real-time feedback on fluoroscopy use, and adherence to optimised imaging protocols.

Overall, radiation safety within orthopaedic trauma practice requires an integrated, continuous approach that combines education, technical optimisation, and regular audit in accordance with IR(ME)R and ALARP principles. Future multi-centre studies with larger sample sizes and case-mix adjustment are warranted to establish benchmark radiation values and evaluate the long-term impact of sustained educational programmes on both patient and staff safety, with potential proposal of national diagnostic reference levels to standardise practice.
